# Band-like transport in small-molecule thin films toward high mobility and ultrahigh detectivity phototransistor arrays

**DOI:** 10.1038/s41467-018-07943-y

**Published:** 2019-01-02

**Authors:** Deyang Ji, Tao Li, Jie Liu, Saeed Amirjalayer, Mianzeng Zhong, Zhao-Yang Zhang, Xianhui Huang, Zhongming Wei, Huanli Dong, Wenping Hu, Harald Fuchs

**Affiliations:** 1 Physikalisches Institut, Westfälische Wilhelms-Universität, Wilhelm-Klemm-Straße 10, 48149 Münster, Germany; 2 Center for Nanotechnology, Heisenbergstraße 11, 48149 Münster, Germany; 3 School of Chemistry and Chemical Engineering, Shanghai Key Laboratory of Electrical Insulation and Thermal Aging, Shanghai Jiao Tong University, Shanghai, 200240 China; 4 Key Laboratory of Organic Solids, Institute of Chemistry, Chinese Academy of Sciences, Beijing, 100190 China; 5 Center for Multiscale Theory and Computation, Westfälische Wilhelms-Universität Münster, Wilhelm-Klemm-Straße 10, 48149 Münster, Germany; 6 State Key Laboratory of Superlattices and Microstructures, Institute of Semiconductors, Chinese Academy of Sciences & College of Materials Science and Opt-Electronic Technology, University of Chinese Academy of Sciences, Beijing, 100083 China; 7 Tianjin Key Laboratory of Molecular Optoelectronic Sciences, Department of Chemistry, School of Science, Tianjin University & Collaborative Innovation Center of Chemical Science and Engineering (Tianjin), Tianjin, 300072 China

## Abstract

With the fast development of organic electronics, organic semiconductors have been extensively studied for various optoelectronic applications, among which organic phototransistors recently emerged as one of the most promising light signal detectors. However, it is still a big challenge to endow organic phototransistors with both high mobility and high light-sensitivity because the low mobility of most organic photoresponsive materials limits the efficiency of transporting and collecting charge carriers. We herein report band-like charge transport in vacuum-deposited small-molecule thin films for organic phototransistor arrays which can be operated at very low dark currents (~10^−12^ A). Both high mobility and excellent optical figures of merit including photosensitivity, photoresponsivity and detectivity are achieved, wherein, unprecedentedly, a detectivity greater than 10^17^ cm Hz^1/2^ W^−1^ is obtained. All these key parameters are superior to state-of-the-art organic phototransistors, implying a great potential in optoelectronic applications.

## Abstract (web summary)

The potential of organic phototransistors to surpass silicon-based devices is limited by the low carrier mobility of organic photoresponsive materials. Here, Ji et al. report high performance organic phototransistors featuring vacuum-deposited small-molecule films with band-like charge transport.

## Introduction

Owing to their ability of capturing and converting incident light into detectable electrical signals, phototransistors have been extensively investigated in the area of optoelectronics, and targeted for many innovative applications such as imaging, optical communication, and biomedical sensing^1,2^. In recent years, organic phototransistors (OPTs) have emerged as one of the most promising light signal detectors, benefiting from the attractive properties of organic semiconductors including low cost, lightweight, compatibility with flexible substrates, ease of large-area processing, and tailorable optoelectronic properties by synthetic methods^3–5^. With a synergistic combination of introducing new materials and optimized preparation technologies, the key figures of merit of OPTs are now comparable and even superior to that of silicon-based phototransistors^6^. However, the low mobility of most organic photoresponsive materials limits the efficiency of transporting and collecting charge carriers, restricting the further development of OPTs^7^. Recently, some single-crystal phototransistors showed promising mobility and light-sensitivity^8^, but precise manipulation of single crystals is still a formidable challenge for large-scale fabrication and application. In comparison, thin-film devices can preferably maximize the advantage of organic semiconductors for more complex integration. For the above reasons, it is highly desirable to develop thin-film OPTs preferably with both high mobility and superior light-sensitivity.

Herein, we report high-performance OPT arrays with vacuum-deposited 2,6-diphenylanthracene (DPA) as organic photoresponsive layers. It is noteworthy that the small-molecule thin-film field-effect transistors show “band-like” charge transport, implying the highly crystalline nature of the thin films. Both high mobility and excellent optical figures of merit are successfully obtained, showing a prospect of high-performance OPTs for both experimental research and practical applications.

## Results

### Characterization of DPA thin films and their TFTs

**Fig. 1:**
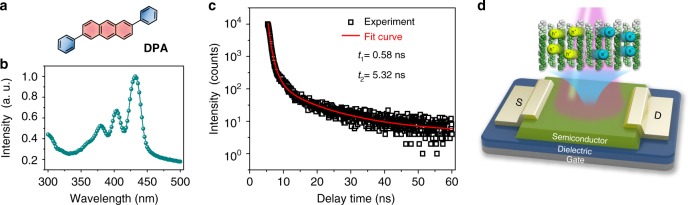
Device structure and optical feature of DPA film. **a** Molecular structure of DPA. **b** UV–vis absorption spectra of DPA film. **c** Spectral-dependent photoluminescence (PL) decay of DPA film. **d** Schematic diagram of OPTs

Compared to polymer materials, small molecule semiconductors have defined molecular structures for convenient synthesis and purification, which is important for the fabrication of high-performance devices^9^. Acenes are typical small molecules that have been extensively studied for organic electronics. It has been verified that extending the π-conjugation system can reduce the reorganization energy and enhance electronic coupling for higher charge carrier mobility^10^, which, however, is normally at the expense of the environmental stability and optoelectronic properties. For example, pentacene-based transistors exhibit a higher mobility than tetracene, but display lower stability and photosensitivity (pentacene, ~10; tetracene, ~10^3^)^11^. In order to achieve a good balance between mobility and stability, we previously reported a simple and unique structured small molecule, DPA (Fig. 1a). The phenyl groups were introduced at 2-, 6- positions of anthracene leading to relative reduction of π conjugation compared with pentacene and the highest occupied molecular orbital (HOMO) energy level of DPA was lowered down to −5.6 eV with an estimated energy band gap of ~3.0 eV, which all helped to improve its stability^12^. Benefitting from a dense herringbone packing motif (Supplementary Fig. 1a) and multi C–H–π interactions (intermolecular distance only 2.84–2.86 Å, Supplementary Fig. 1b), a charge carrier mobility of its single crystal up to 34 cm^2^ V^−1^ s^−1^ was achieved^13^, which was comparable to the best performance of single-crystalline pentacene^14^. Moreover, anthracene with strong fluorescence was used as the semiconducting core, contributing to a photoluminescence quantum yield of DPA of up to 41.2%^13^. As shown in Fig. 1b, c, DPA thin film exhibits strong absorption in the UV–vis range and a three orders of magnitude longer exciton lifetime (~1.5 ns) than pentacene (~1 ps)^11^, indicating its great potential for applications in optoelectronics, such as OPTs (Fig. 1d).

**Fig. 2:**
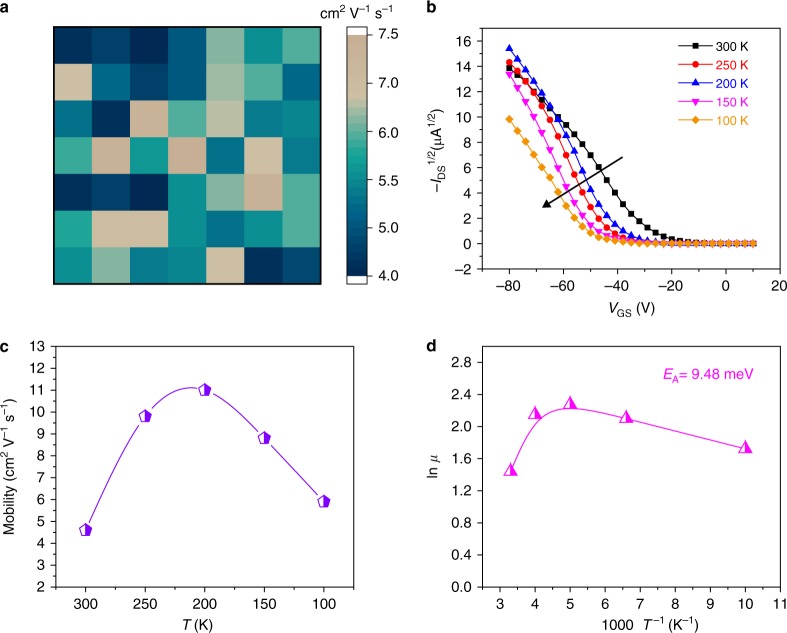
Electrical properties of DPA-based TFTs. **a** Distribution of TFT mobilities. **b** Square root of drain current (−*I*_DS_^1/2^) versus gate voltage (*V*_GS_) measured at different temperatures. **c** Temperature dependence of mobility. **d** ln(*μ*) versus 1000/*T*

Highly ordered and highly crystalline (X-ray diffraction measurement, Supplementary Fig. 2) 20 nm DPA thin-film field-effect transistors (TFTs) arrays (7 × 7) were fabricated on octadecyltrichlorosilane (OTS)-treated SiO_2_(300 nm)/Si substrate with gold as top-contact source/drain electrodes (Supplementary Fig. 3). Different thicknesses (5, 10, 15, and 20 nm) of DPA thin films were deposited onto OTS/SiO_2_(300 nm)/Si substrate and a layer-by-layer grown mode was clearly observed during the film growth process from 5 to 20 nm (Supplementary Fig. 4), which was consistent with the previous work^12^. The *d*_001_ spacing was calculated to be ~1.8 nm from the (001) diffraction pattern peak (2*θ* = 4.88) of the DPA film (Supplementary Fig. 2), which was very close to the length of an individual DPA molecule^12^. This indicated that DPA molecules preferentially grew perpendicularly on the OTS surface, which offered a favorable charge transfer channel at the semiconductor–dielectric interface^15^. As a result, all the devices exhibited high mobility values ranging from 4 to 7.5 cm^2^ V^−1^ s^−1^ (Fig. 2a) and outstanding operating stability in the dark with ON–OFF ratios of 10^7^–10^8^ and switching cycle of >3000 times at ambient conditions (Supplementary Fig. 5a). The typical transfer and output characteristics are shown in Supplementary Fig. 6. The DPA thin-film transistors were also tested in the vacuum with the temperature lowered down from 300 to 100 K. Figure 2b depicts representative transfer curves at varied temperatures with the square root of source/drain current (−*I*_DS_) plotted against the gate voltage (*V*_GS_), showing the threshold voltage (*V*_th_) shifted to more negative values (Supplementary Fig. 7) with decreasing temperature. This phenomenon is commonly observed from other organic field-effect transistors^16–18^, indicating the gate voltage fill up low-mobility trap states^19^. Correspondingly, the temperature dependence of the mobility is further shown in Fig. 2c. Interestingly, an increase of the mobility (from 4.6 to 11 cm^2^ V^−1^ s^−1^) was recorded with decreasing temperature (from 300 to 200 K). This negative temperature coefficient of hole mobility (d*µ*/d*T* < 0) demonstrates that band-like transport exists^19^. It is worth emphasizing that most of the reported band-like transport occurs in single crystals due to their intrinsic order^16–19^. We can therefore infer that the observed band-like transport very likely indicates a high degree of order present in vacuum-deposited DPA thin films, as corroborated by the X-ray measurements. When the temperature was below the inflection point (200 K, Fig. 2c), the charge carriers became thermally activated, which was dominated by shallow traps^20,21^ and resulted in a decrease of mobility to 5.9 cm^2^ V^−1^ s^−1^ at 100 K. According to *μ* = *μ*_0_exp(−*E*_A_/*k*_B_*T*), where *E*_A_ is the activation energy and *k*_B_ is the Boltzmann constant, a value of about 9.48 meV for *E*_A_ was calculated (Fig. 2d). Thus, the low activation energy of thin-film DPA indicated a low degree of energetic disorder at the interface^22,23^ between thin-film DPA and OTS for the further study of photoelectric properties.

### Charge transfer mechanism and performance of the OPTs

**Fig. 3:**
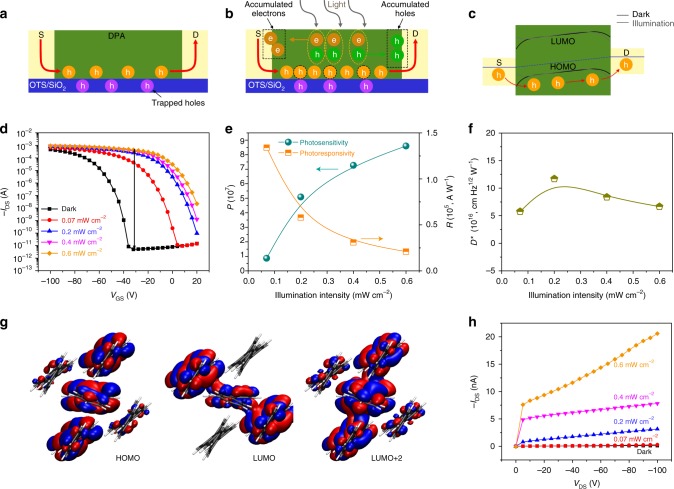
Charge transfer mechanism and performance of the phototransistor. Charge transfer mechanism in dark (**a**) and under illumination (**b**). **c** Energy band diagram of electrode/semiconductor interface in dark and under illumination. **d** Transfer characteristics of DPA-based phototransistor measured under different illumination intensities in the air. **e** *P* and *R* as a function of illumination intensity. **f** D* as a function of illumination intensity. **g** Calculated molecular orbitals describing the first electronic excitation (*E*_vert_ = 388.67 nm; *f* = 0.1331) at the TD-DFT level. **h** −*I*_DS_ at various illumination intensities (*V*_GS_ = 0 V)

High mobility values demonstrate high efficiency of transporting and collecting charge carriers, normally contributing to higher photosensitivity and photoresponsivity in the OPTs (Table 1). For p-channel OPTs operating in the dark, some charge carriers (holes) will be trapped between the active layer and dielectric layer by the interface defects (Fig. 3a), and these trapped holes will increase the turn-on gate voltage required for transistors, shifting the threshold voltage towards a higher value. When the device is under illumination and the photovoltaic mode is dominant, the photogenerated electrons/holes in the conductive channel will move and accumulate around the source/drain electrodes (Fig. 3b) driven by the inner electric field^24^, which leads to band bending^25^ in the semiconductor (Fig. 3c). As a result, the potential barrier for the injection of holes into the source electrode is lowered^26^, and there will be more holes involved in the charge transport process (Fig. 3b) under the same driving voltage compared to the situation in the dark, thus increasing the photocurrent and inducing a positive shift of the threshold voltage. Typical transfer characteristics of the phototransistor in dark and under illumination are shown in Fig. 3d. Moreover, the OPTs showed outstanding operating stability under the illumination intensity of 0.6 mW cm^−2^ with ON–OFF ratios of 10^6^–10^7^ and switching cycle of >3000 times at ambient conditions (Supplementary Fig. 5b). From the transfer curve in dark, a relatively high threshold voltage (*V*_th_) (~−53 V) was recorded, which could be attributed to both the contact resistance (a ~0.4 eV mismatch between the HOMO energy level of DPA (5.6 eV) and the work function of gold (5.2 eV)), and the relatively high density of traps (5.94 × 10^12^ cm^−2^) at the dielectric interface increasing the turn-on voltage of the transistors. As a consequence, the phototransistor in the off-state exhibited a low dark current of only several pA. With an increase of illumination intensity (from 0 to 0.6 mW cm^−2^), a large shift of the threshold voltage (*V*_th_) was observed (Supplementary Fig. 8) with the mobility increased to 12 cm^2^ V^−1^ s^−1^ (Supplementary Fig. 9). The large shift of the *V*_th_ (~+63 V) demonstrated high density of trapped photogenerated carriers (Δ*N*_trap_ = Δ*V*_th_*C*_ox_/*q*^27^, where *q*, *C*_ox_, and Δ*V*_th_ are elementary charge, capacitance of the dielectric layer, and threshold voltage shift, respectively) at the dielectric/semiconductor interface^28^. The increased mobility indicated a large number of photogenerated carriers formed the conductive channel to improve charge transfer. In addition, the output currents of DPA-based OPTs under different illumination intensities increased accordingly (Supplementary Fig. 10), also indicating that more charge carriers were generated^29^. Consequently, a high number of photogenerated carriers and more efficient charge trapping jointly led to a high photoresponse^30^. Supplementary Fig. 11a shows the photosensitivity (*I*_illumination_/*I*_dark_, *P*) as a function of gate-source voltage (*V*_GS_) with the best value reaching as high as ~8.5 × 10^7^ (Fig. 3e) at *V*_GS_ ~ −30 V under the illumination intensity of 0.6 mW cm^−2^.

**Table 1:** Comparison of current work with representative phototransistors based on organic semiconductors

Semiconductor	Mobility (cm^2^ V^−1^ s^−1^)	Photosensitivity	Photoresponsivity (A W^−1^)	Detectivity (cm Hz^1/2^ W^−1^)	Light density (mW cm^−2^)	Ref.
BBDTE (SC)	1.62	10^5^	9.8 × 10^3^	N/A	0.037	^8^
Me-ABT (SC)	1.66	10^4^	1.2 × 10^4^	N/A	0.03	^35^
TFT-CN (SC)	1.36	5 × 10^5^	9 × 10^4^	6 × 10^14^	0.179	^36^
A-EHDTT (SC)	1.2–1.6	1.4 × 10^5^	1.4 × 10^4^	N/A	0.0014	^37^
PY-4(THB) (SC)	0.7	1.2 × 10^6^	2 × 10^3^	N/A	0.0056	^38^
p-DTS(FBTTh_2_)_2_ (SC)	1.8	10^3^–10^4^	3 × 10^3^	N/A	7	^39^
DNTT (TF)	N/A	8.1 × 10^4^	1.7 × 10^4^	2 × 10^14^	1	^31^
Spiro-DPSP (TF)	1.3 × 10^−6^	5 × 10^2^	1	N/A	0.19	^40^
6T (TF)	0.09	1.3 × 10^3^	1.5–2.4	N/A	1.5	^41^
ABT(TF)	0.4	800	1000	N/A	0.03	^42^
DPP-DTT/PCBM (TF)	N/A	5 × 10^4^	350	5.7 × 10^13^	89	^43^
DPP-DTT/PCBM (TF)	0.14 p/0.06 n	3 × 10^4^	8 × 10^5^	3 × 10^12^	57	^44^
F_16_CuPc (TF)	5.3 × 10^−4^	22	1.5 × 10^−3^	N/A	5.6	^45^
P3HT (TF)	0.01-0.07	3.8 × 10^3^	245	N/A	51	^46^
TIPS-Pentacene (TF)	0.02	10^6^–10^7^	N/A	N/A	9–13	^47^
DPA (TF)	7.5^a^/12^b^	8.5 × 10^7^	1.34 × 10^5^	1.2 × 10^17^	0.07–0.6	This work

*SC* single crystal, *TF* thin film ^a^Dark ^b^Illumination

The photoresponsivity (*R*) and detectivity (D*) are other key figures of merit used to evaluate the phototransistor performance. *R* can be expressed as

1 $$R = I_{\rm illumination}(P_iS)^{ - 1}$$

where *P*_*i*_ is the incident light intensity and *S* is the area of the illuminated channel. Figure 3e and Supplementary Fig. 11b depict the dependence of *R* with illumination power intensity and *V*_GS_, respectively. All the devices could achieve photoresponsivity higher than 10^4^ A W^−1^ with the best value up to 1.34 × 10^5^ A W^−1^ at the *V*_GS_ of ~−100 V with an illumination intensity of 0.07 mW cm^−2^, which proves their strong ability to convert light into electric current. The specific detectivity (D*) can be written as^31^

2 $$D^ \ast = R\left( {S\Delta f} \right)^{1/2}(i_n)^{ - 1}$$

where Δ*f* is the operation bandwidth and *i*_*n*_ is the measured noise current. Assuming that the major contribution is the shot noise from dark current, D* is then given by^32^

3 $$D^ \ast = RS^{1/2}(2{\mathrm e}\,I_{\rm Dark})^{ - 1/2}$$

An unprecedented D* value of 1.2 × 10^17^ cm Hz^1/2^ W^−1^ was obtained (Fig. 3f and Supplementary Fig. 11c), which can be attributed to the low dark current (~10^−12^ A), high photoresponsivity (*R*) and the small area (7.2 × 10^−5^ cm^2^) of the illuminated channel as stated in Eq. (3). This superior detectivity also indicates that the devices would be very powerful for detecting incident weak light signals. To the best of our knowledge, these essential key parameters, including *P*, *R*, and D*, are superior to state-of-the-art OPTs (Table 1)^4,5^ and are among the best of all previously reported phototransistors to date^3,33–48^. We also tested the stability of the devices and found even after 4 months stored in the air (Supplementary Fig. 12), the best values of *P*, *R*, and D* could still reach 4.4 × 10^7^, 1.1 × 10^5^ A W^−1^, and 0.98 × 10^17^ cm Hz^1/2^ W^−1^, respectively, which indicated high stability of the DPA-based OPTs.

### Time-dependent density functional theory (TDDFT) calculations

In order to understand the photophysical properties, time-dependent density functional theory (TDDFT) calculations were performed. Due to highly ordered and highly crystalline DPA thin film deduced from its high charge mobility and “band-like” charge transport property, we thus first optimized, based on the single crystal data (Supplementary Fig. 1a), the structure and packing mode of DPA (Supplementary Fig. 1b). The structure obtained by periodic DFT calculations (Supplementary Fig. 1c) is in good agreement with the experimental result. To get insight into the electronic properties of the molecular systems after photo-excitation, the electronic excitation was subsequently calculated by using a model system consisting of 5 DPA molecules. Based on these calculations, the alteration of the electronic distribution upon photo-excitation can be extracted. The detailed analysis of the first excited state revealed that this electronic transition is mainly associated with a transition from the HOMO to the lowest unoccupied molecular orbital (LUMO) and LUMO + 2 (Fig. 3g). Comparing these molecular orbitals before and after excitation provides a fingerprint of the photo-induced redistribution of electrons and reveals an increase of electron density between the DPA molecules after electronic excitation. This enhanced electronic overlap between adjacent molecules promotes the charge carrier mobility in the system. The TDDFT calculations are consistent with the improved performance of the DPA system under illumination and allow rationalizing the band-like transport properties.

### Grain boundaries (GB) effect on the performance of the OPTs

In order to further verify that efficient carrier transport is a prerequisite for high light-sensitivity, we also studied the grain boundaries (GB) effect on the performance of the OPTs and adopted another two thicknesses (10 and 30 nm) of DPA active layers. As for 10 nm DPA film, the OTS/SiO_2_/Si substrate was not fully covered, leading to higher density of GB than that of 20 nm film. When the deposited film was thicker than 20 nm (e.g., 30 nm), it was found that surface aggregation generally resulted in the growth of more GB (Supplementary Fig. 13). Typical transfer characteristics of the phototransistors based on 10 and 30 nm in dark and under illumination are shown in Supplementary Fig. 14. As shown in Supplementary Fig. 15a, the mobilities of all the devices were enhanced under illumination, and it was found that the mobility was mainly affected by the density of GB, rather than the film thicknesses (*μ*_20nm_ > *μ*_30nm_ > *μ*_10nm_). This phenomenon can be explained by the trapping of charge carriers by the GB during carrier transport, resulting in a decrease of the mobility. In addition, the corresponding output current also depended on the efficient carrier transport (*I*_20nm_ > *I*_30nm_ > *I*_10nm_) (Supplementary Fig. 16). *P* and *R* showed the same trend as that of the mobility (*P*_20nm_ > *P*_30nm_ > *P*_10nm_, Supplementary Fig. 15b and *R*_20__nm_ > *R*_30nm_ > *R*_10nm_, Supplementary Fig. 15c), which further confirmed that higher mobility would contribute to higher photosensitivity and photoresponsivity.

### The performance of the photodetectors

Owing to their excellent light-sensitivity, DPA-based OPTs are promising for the use of high-performance photodetectors. As shown in Fig. 3h, light irradiation can act as an independent parameter for regulating the output current of the OPTs at different illumination intensities (*V*_DS_ = −100 V, *V*_GS_ = 0 V). The photocurrent was enhanced gradually with the increase of illumination intensity (from 0 to 0.6 mW cm^−2^) and the device contact-resistance correspondingly decreased (Supplementary Fig. 17), which can be attributed to the lowered injection barrier induced by light irradiation. The output current generated at fixed illumination was stable with the time-dependent photocurrent (0.6 mW cm^−2^, *V*_GS_ = 0 V) shown in Supplementary Fig. 18. Subsequently, we monitored the current between the source and drain (*I*_DS_, *V*_DS_ = −100 V, *V*_GS_ = 0 V) while regularly turning the light on and off. As shown in Supplementary Fig. 19, the OPT device exhibited photoswitching time of *τ*_rising_ = ~1 s and *τ*_decay_ = ~1 s, and the ON–OFF ratio was depended on the illumination intensity (Supplementary Fig. 20). Compared with inorganic phototransistors^49–51^, the relatively long response time of DPA-based OPTs can be ascribed to trapped photogenerated charge carriers at the organic semiconductor/dielectric interface and the slow nature of the recombination of generated carriers^5,52,53^. An example of three consecutive ON–OFF cycles is shown in Supplementary Fig. 21.

**Fig. 4:**
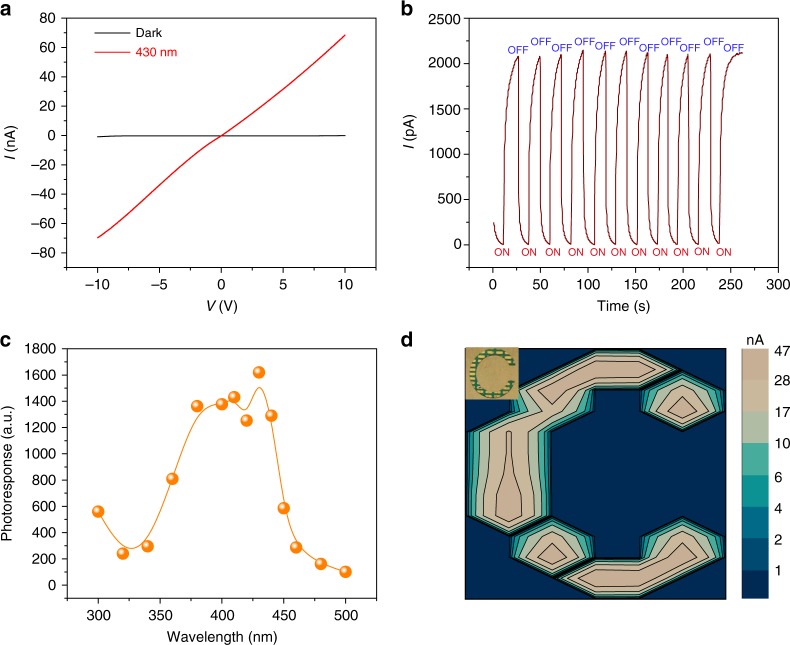
Performance of the two-terminal DPA-based photodetector. **a** The typical *I*–*V* curve of the two-terminal device in dark and under illumination at 430 nm (0.4 mW cm^−2^). **b** Photo-response measurements of the device in dark and under illumination (bias voltage, 10 V). **c** *I*_illumination_/*I*_dark_ as a function of time under illumination. **d** Current mapping of the 6 × 11 imaging matrix under illumination with “C” type mask (1.5 cm × 1.5 cm) on top

A two-terminal photodetector was also fabricated and according to the absorption spectrum, the photodetector was irradiated using monochromatic light at 430 nm (0.4 mW cm^−2^). The resistance of the device was as high as 1.1 × 10^12^ Ω in the dark at a bias voltage of 10 V and it quickly dropped to 1.5 × 10^8^ Ω when the light source was turned on (Fig. 4a). With the light on and off, consecutive ON–OFF cycles (ON–OFF ratio up to ~10^3^) were recorded (Fig. 4b) with photoswitching time of *τ*_rising_ = ~0.4 s and *τ*_decay_ = ~0.4 s, respectively (Supplementary Fig. 22). Figure 4c shows the typical photoresponse (*I*_illumination_/*I*_dark_) with irradiation wavelengths ranging from 300 to 500 nm. The current spectrum agreed well with the optical absorption curve, which further confirmed the photoresponse of the detector at 430 nm wavelength originated from the light absorption of the DPA layer. Moreover, a 6 × 11 imaging matrix on a silicon wafer was fabricated. The current signals shielded by the shadow mask with a hollowed symbol “C” (Fig. 4d) were in the 10^−10^ A level, while the output currents from the light exposed areas were kept at around 10^−8^ A. As shown in Fig. 4d, by recording the current of the matrix, a spatial mapping of the characters can be presented with high accuracy. All the above evidence proved the excellent optoelectronic properties of DPA molecules.

## Discussion

In conclusion, high-performance and high-stability phototransistors based on small-molecule DPA were investigated. The OPTs not only have high mobility (12 cm^2^ V^−1^ s^−1^ under illumination), but also show outstanding photoresponsivity (1.34 × 10^5^ A W^−1^), detectivity (1.2 × 10^17^ cm Hz^1/2^ W^−1^) and *I*_illumination_/*I*_dark_ ratio (8.5 × 10^7^). Simultaneously, two-terminal photodetectors irradiated under 430 nm illumination show ON–OFF ratios up to 2 × 10^3^ in the air. All the above values are superior to state-of-the-art OPTs and are among the best of all previously reported phototransistors. We believe the small-molecule DPA offers great opportunity toward high-performance OPTs and their arrays for both fundamental research and practical applications.

## Methods

### Device fabrication and characterization

Bottom-gate top-contact DPA thin film transistors were fabricated by the following procedures: SiO_2_/Si substrates used in the study were successively cleaned with deionized water, acetone, pure ethanol, piranha solution (H_2_SO_4_:H_2_O_2_ = 7:3), deionized water, isopropanol, and then dried with nitrogen. The surface of SiO_2_/Si substrate was treated with O_2_ plasma (50 W, 1 min). Here, plasma treatment was carried out using Gala Instrument Prep2; treatment of SiO_2_/Si wafer with OTS was then carried out by conventional vapor deposition method at a vacuum chamber (0.1 Pa) at 120 °C for 2 h; the substrate was transferred to a vacuum chamber and thin films of 20 nm DPA was deposited on OTS treated SiO_2_/Si at a substrate temperature of 50 °C and a deposition speed of 0.05 Å s^−1^; 30 nm Au as source/drain electrodes were deposited on the DPA surface with metal mask with a deposition speed of 1 Å s^−1^. The morphology of DPA was characterized by atomic force microscopy (AFM) using a Nanoscope IIIa instrument (USA). X-ray diffraction (XRD) of the DPA layer was recorded on a D/max2500 with a Cu Kα source (*k* = 1.541 Å). The electrical characteristics of the DPA TFTs were measured at room temperature in air and in the vacuum by using a Keithley 4200 SCS semiconductor parameter analyzer and a Micromanipulator 6150 probe station. The mobility was extracted from the saturation region by using the equation of *I*_DS_ = (*W*/2*L*)*C*_*i*_*µ*(*V*_G_−*V*_T_)^2^.

### Computational details

The VASP code was used for all periodic density functional theory (DFT) calculations^54^. The unit cell consisting of two DPA molecules was optimized (both lattice constant and molecules) applying the projector-augmented-wave-based pseudopotentials^55,56^ together with the PBE GGA-type functional^57^. Dispersion effect was accounted in the framework of the DFT-D3 correction method developed by Grimme et al.^58^. The plane-wave cutoff for the wave functions was 800 eV and ionic relaxations were carried out until all forces were smaller than 20 meV/A. The analysis of the electronic properties was performed at the CAM-B3LYP level together with the 6-31 G** basis set as implemented in the Gaussian package^59^ using the non-periodic model system as described in the main text.

## Supplementary Information

Supplementary Information

## Data Availability

The authors declare that the data supporting the findings of this study are available from the corresponding authors upon reasonable request.
