# Supplementary material for: Band-like transport in small-molecule thin films toward high mobility and ultrahigh detectivity phototransistor arrays
Source: Nat Commun. 2019 Jan 2;10:12. doi: 10.1038/s41467-018-07943-y (PMC6315033; doi:10.1038/s41467-018-07943-y)
Supplement: Supplementary file 1 — Supplementary Information [file 41467_2018_7943_MOESM1_ESM.pdf]

Supplementary Information

Band-like Transport in Small-Molecule Thin Films toward High Mobility and Ultrahigh Detectivity Phototransistor Arrays

Ji et al.

Supplementary Figures

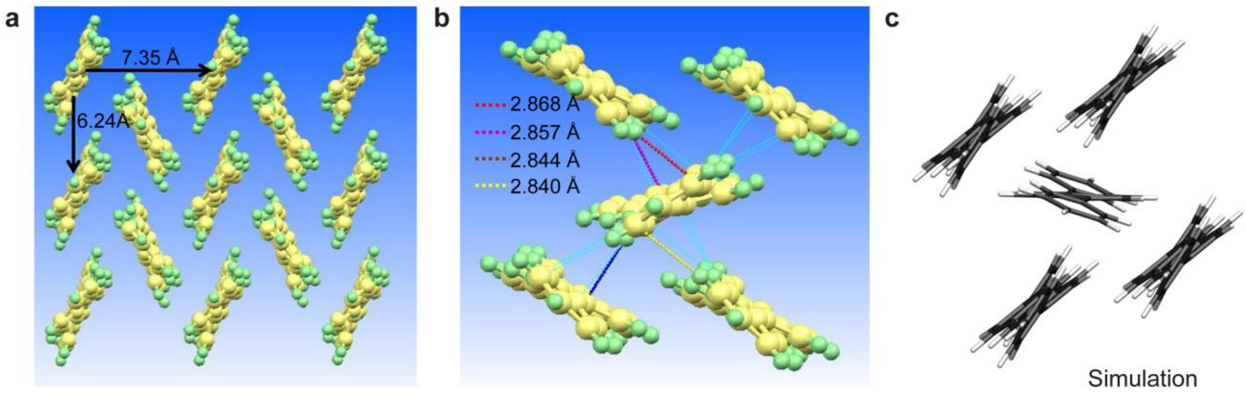

Supplementary Figure 1| Packing structure of DPA and TD-DFT calculations. DPA shows a typical densely herringbone packing and multi C-H-π interactions (only 2.84-2.86 Å) are observed between every DPA molecule and its nearest four neighbor molecules, which indicates DPA would afford high charge carrier mobility. (a) Herringbone packing of DPA. (b) Multi C-H-π interactions in DPA. (c) The optimized geometry and packing of DPA from the periodic DFT calculations.

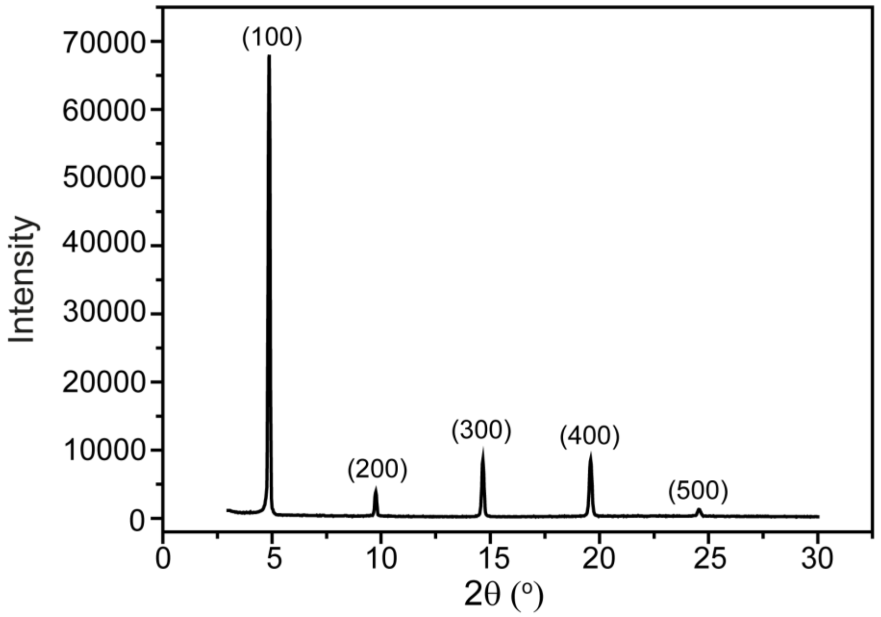

Supplementary Figure 2| XRD patterns of DPA films. XRD patterns of 20 nm-thickness DPA films grown on OTS/SiO_2/Si surface. X-ray diffraction measurements show a series of peaks assignable to (*h*00) reflections, indicating highly ordered and highly crystalline of this film. The peaks at 2θ = 4.88°, 9.76°, 14.66°, 19.6°, and 24.56° are assigned to the (100), (200), (300), (400), and (500) crystal planes of DPA, respectively.

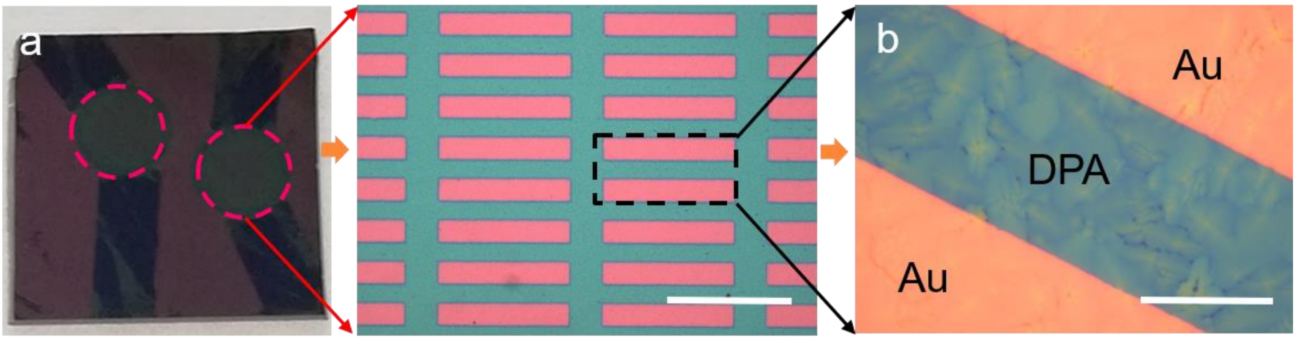

Supplementary Figure 3| Optical microscopy images of device arrays. (a) The OPT device arrays on 1.5 × 1.5 cm wafer and enlarged image of OPT arrays with working area of 30 µm × 240 µm. Scale bar, 240 µm. (b) Individual OPT device. Scale bar, 24 µm.

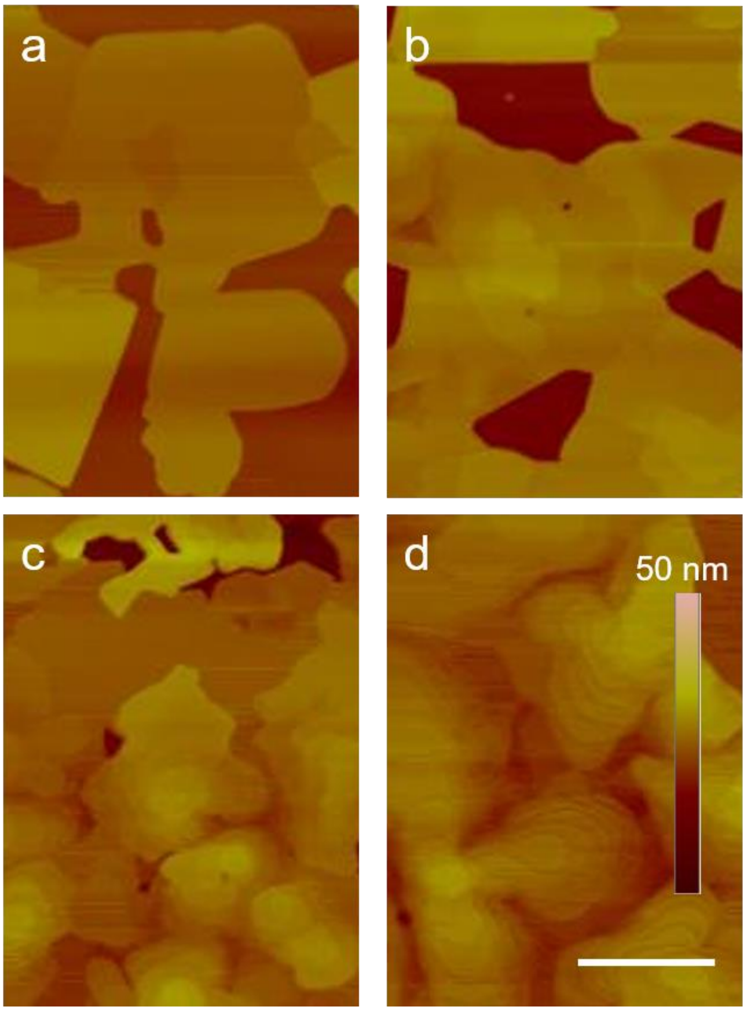

Supplementary Figure 4| AFM images of DPA films. AFM image of (a) 5 nm, (b) 10 nm, (c) 15 nm, (d) 20 nm DPA films on the OTS/SiO_2/Si substrate and layer-by-layer growth mode was clearly observed. Scale bar 1 µm.

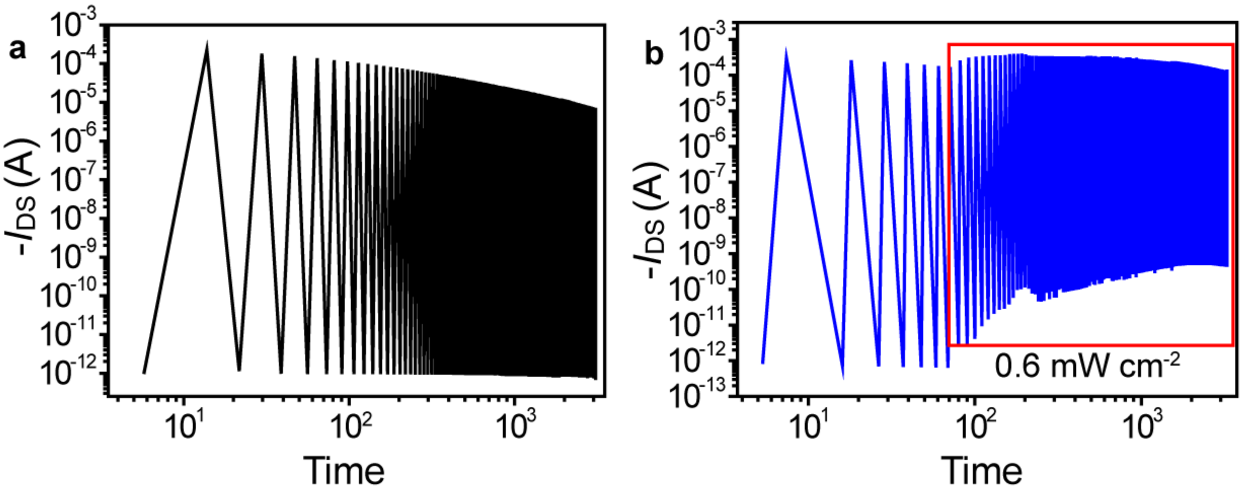

Supplementary Figure 5| The operating stability characterization of DPA OTFTs. (a) The drain current could be switched for more than 3000 times, and ON-OFF ratio still remained above 10^7 in the dark. (b) The drain current could be switched for more than 3000 times, and ON-OFF ratio still remained above 10^6 under illumination of 0.6 mW cm^{-2}.

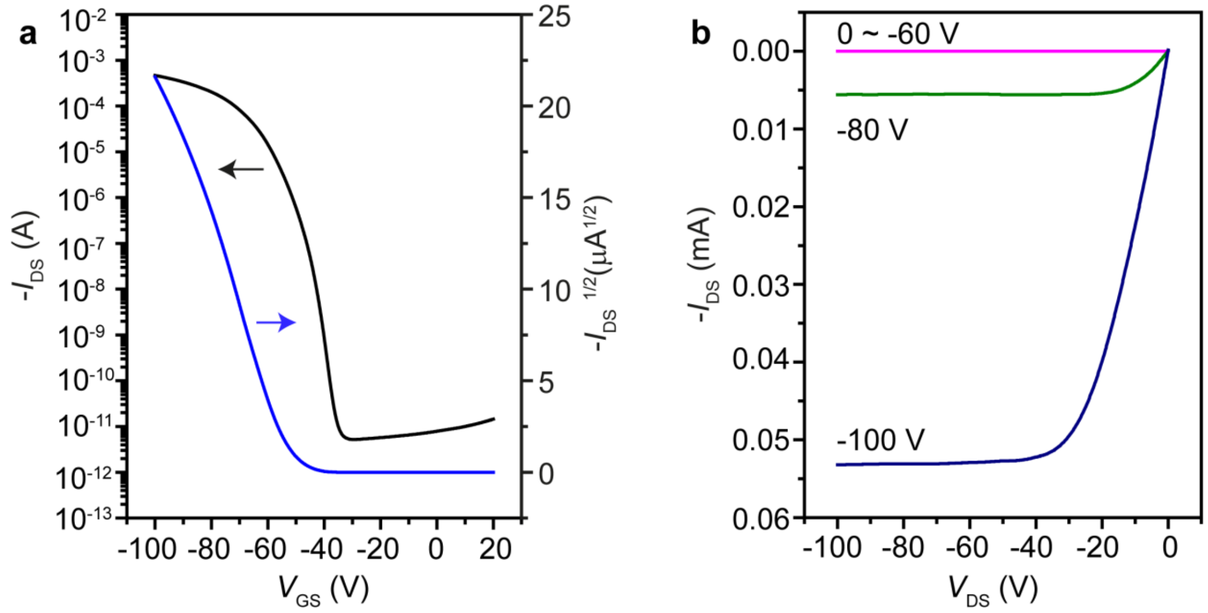

Supplementary Figure 6| The transfer and output characterization of DPA OTFTs. Typical transfer curve (a) and output (b) of the OTFT with 20 nm DPA and a channel dimension of W = 240 μm, L = 30 μm.

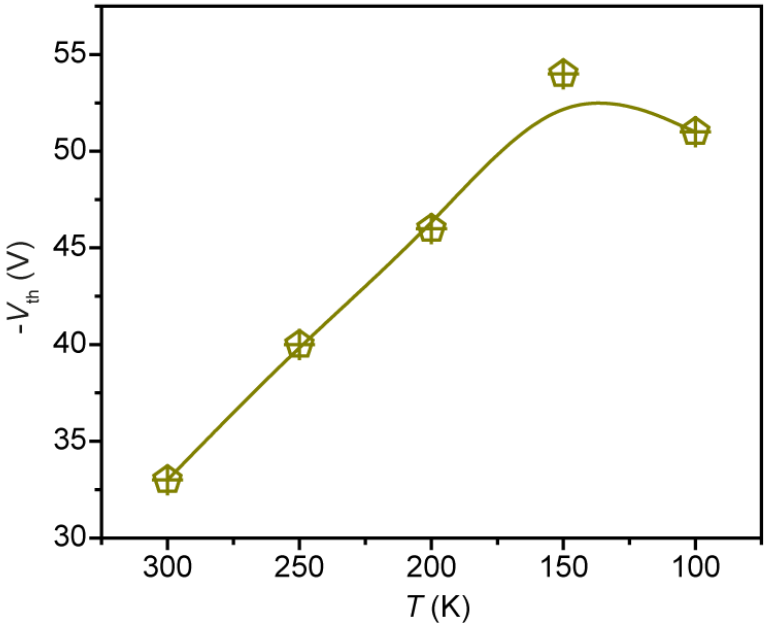

Supplementary Figure 7| The measurement of V_{th} in the vacuum. V_{th} as a function of temperature (T).

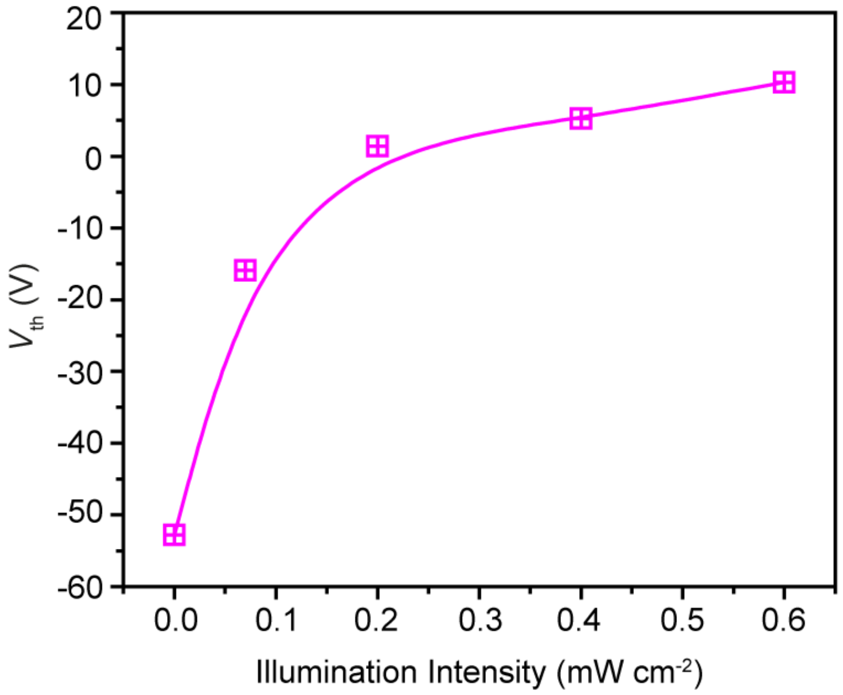

Supplementary Figure 8| The measurement of V_{th} in the air. V_{th} as a function of illumination intensity. It was noted that the transistor showed a relatively high threshold voltage (-53 V) in dark. With the increase of illumination power intensity (from 0 to 0.6 mW/ cm^2), the threshold voltage shifted to more positive values (from ~ -53 V to ~ 10 V).

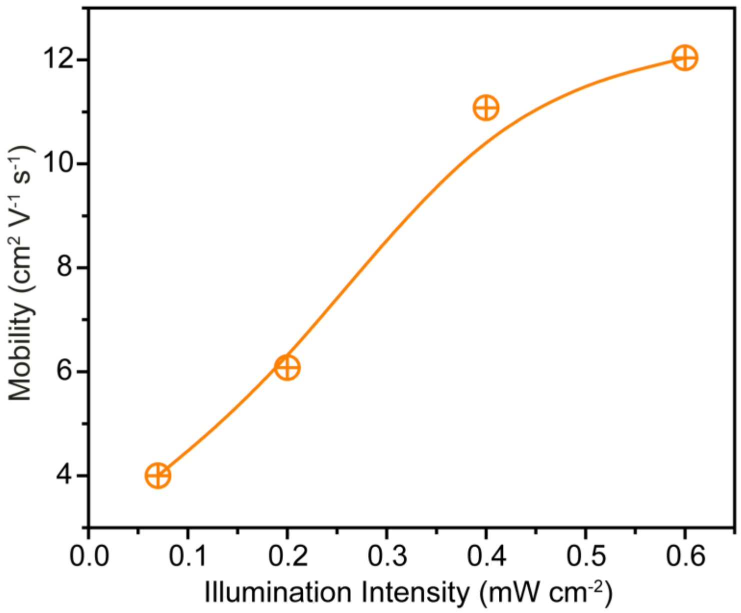

Supplementary Figure 9| The measurements of the charge carrier mobility under illumination.

Mobility as a function of illumination intensity.

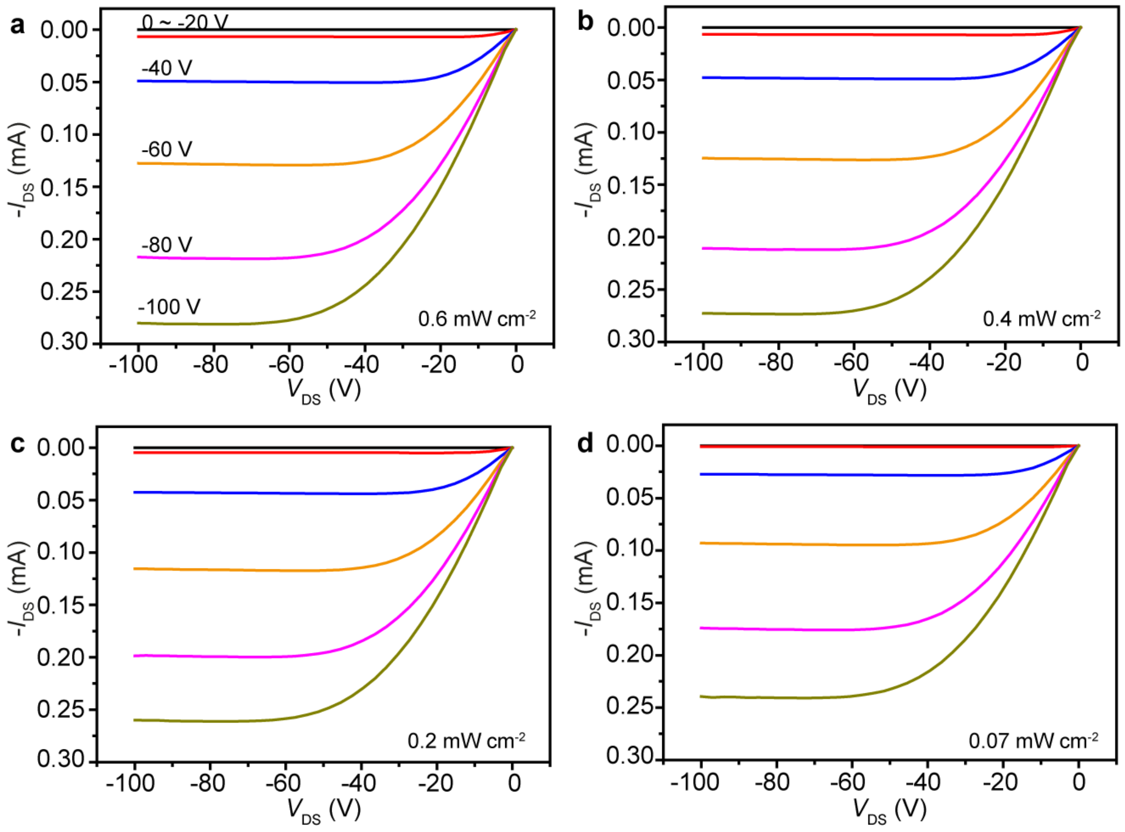

Supplementary Figure 10| The measurements of the output curve under illumination. Typical output of the phototransistor with 20 nm DPA under different illumination intensity. (a) 0.6 mW cm^{-2}. (b) 0.4 mW cm^{-2}. (c) 0.2 mW cm^{-2}. (d) 0.07 mW cm^{-2}. Supplementary Fig. 10 showed the output characteristics of DPA-based OPT under different illumination power intensity. Observably, source-drain current was controllable and could be obtained much higher under illumination than that under dark (Supplementary Fig. 6b) at the same conditions.

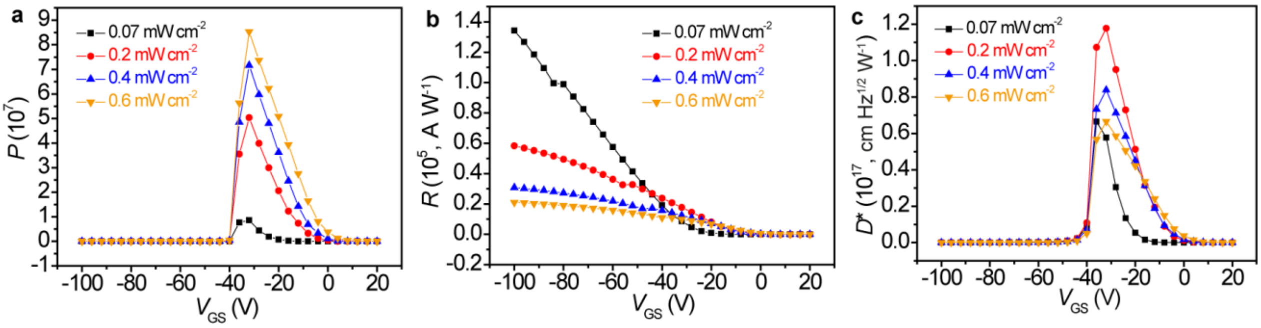

Supplementary Figure 11| The effect of V_{GS} modulation on device performance. (a) *P*, (b) *R* and (c) *D** as a function of V_{GS} under different illumination densities.

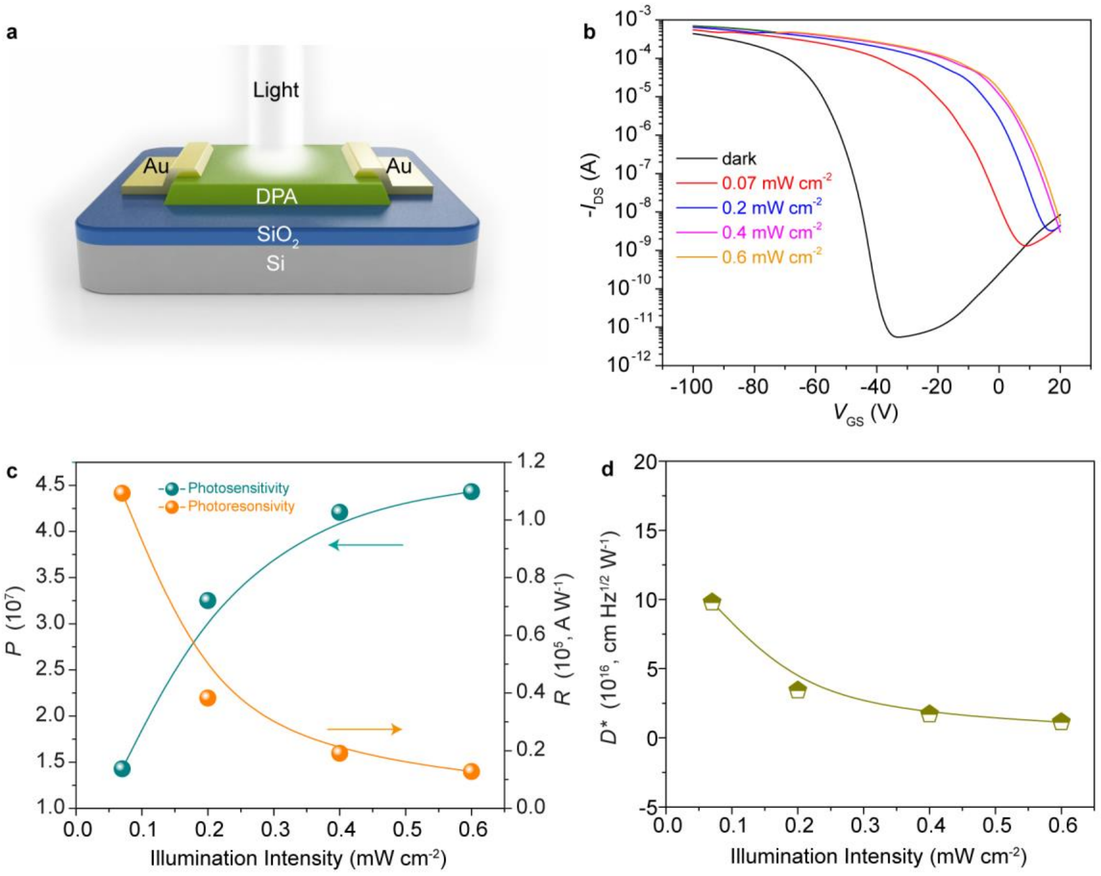

Supplementary Figure 12| The performance of the phototransistor after four months. (a) Schematic diagram of DPA-based phototransistor. (b) Transfer characteristics measured under different illumination intensity in the air. (c) *P* and *R* as a function of illumination intensity. (d) *D** as a function of illumination intensity.

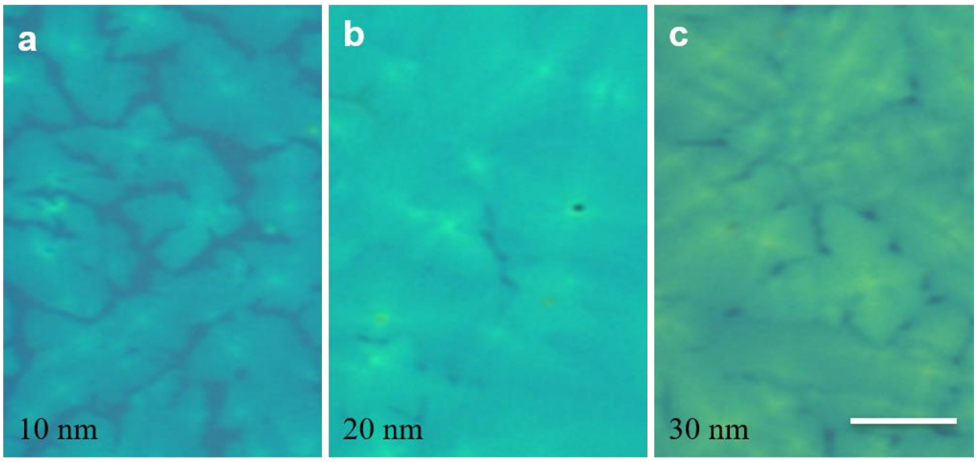

Supplementary Figure 13| Optical microscope images of DPA films. The optical microscope images of deposited DPA films with different thicknesses: (a) 10 nm, (b) 20 nm and (c) 30 nm. Scale bar 10 µm.

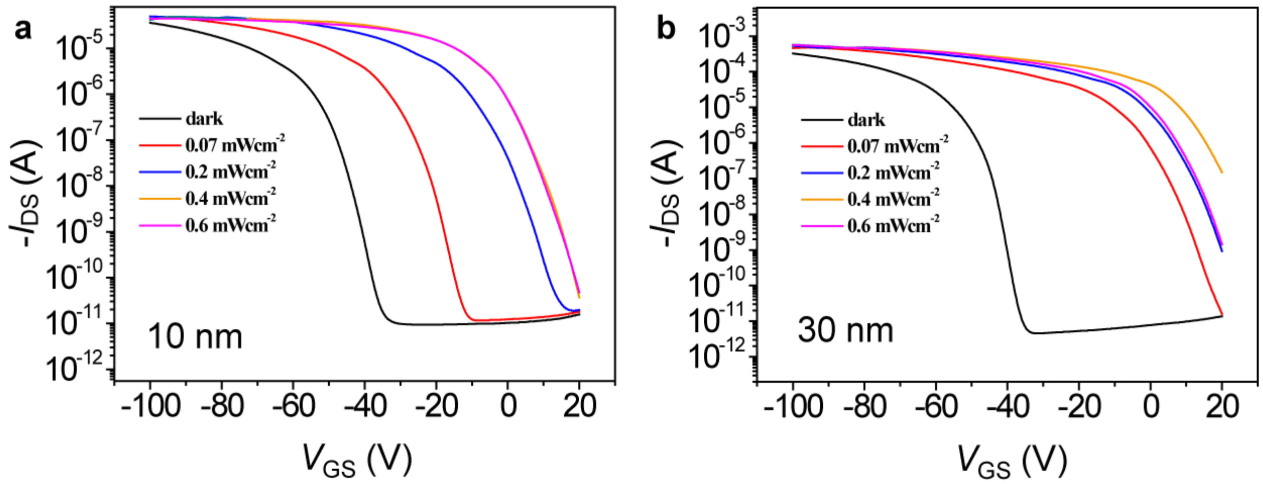

Supplementary Figure 14| Transfer characteristics of the phototransistors. Transfer characteristics of the phototransistors measured under different illumination intensities in the air (from 0 to 0.6 mW cm^{-2}) based on (a) 10 nm and (b) 30 nm active layers.

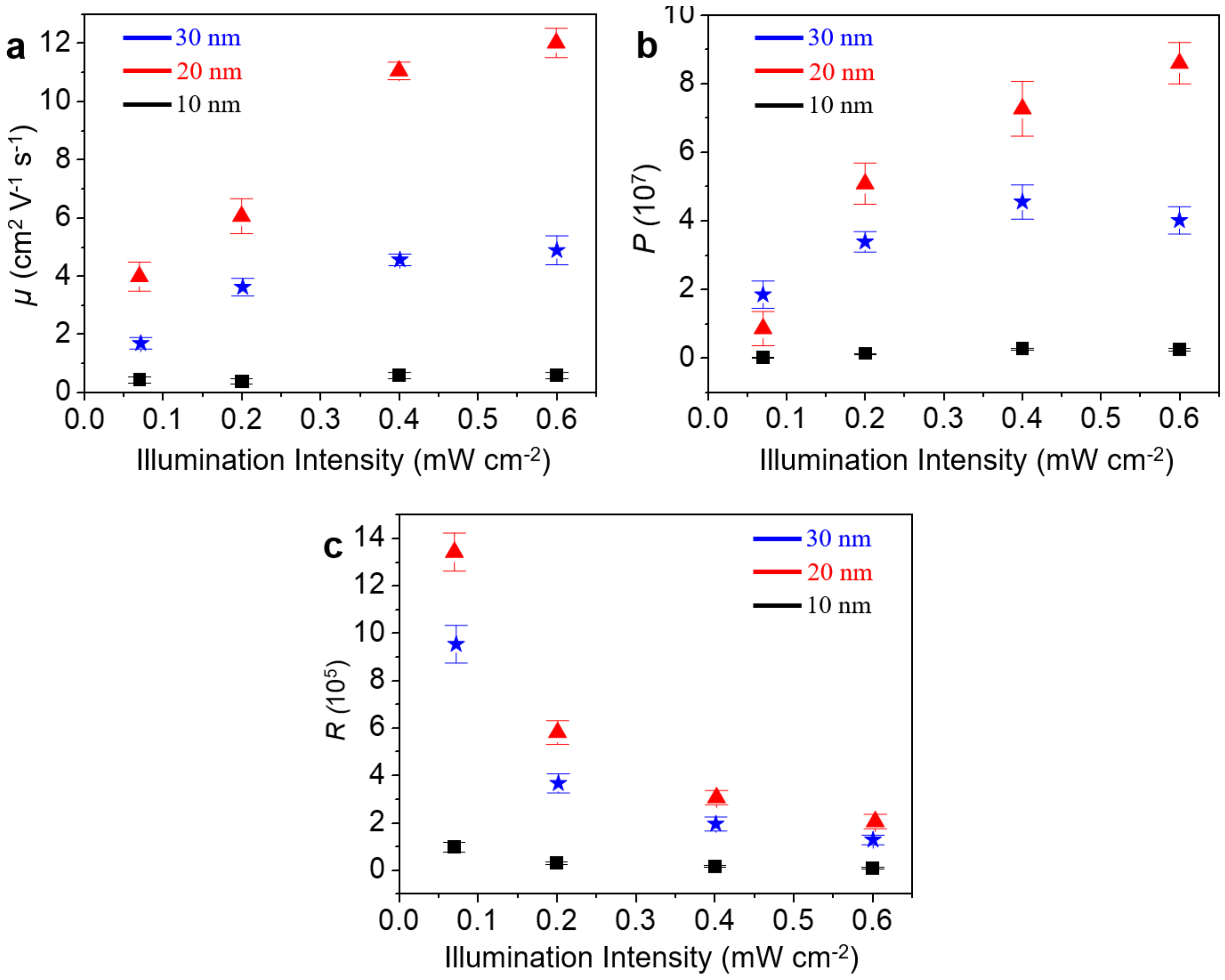

Supplementary Figure 15| Comparative experiments based on different thicknesses of DPA. (a) Mobility (μ) as a function of illumination intensities. (b) P as a function of illumination intensities. (c) R as a function of illumination intensities. The error bars show the standard error of the mean.

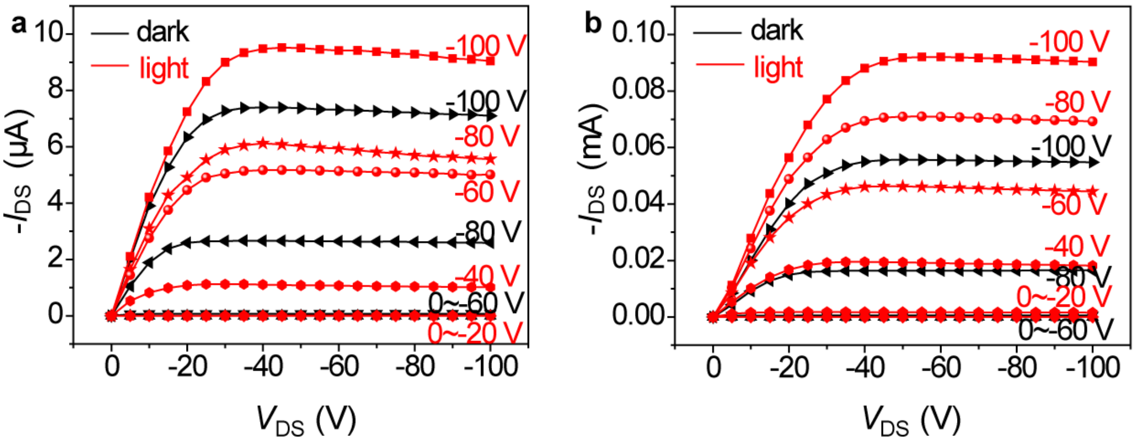

Supplementary Figure 16| Output characteristics of the phototransistors. Typical output curves of the phototransistors with (a) 10 nm and (b) 30 nm DPA active layers in the dark and under illumination.

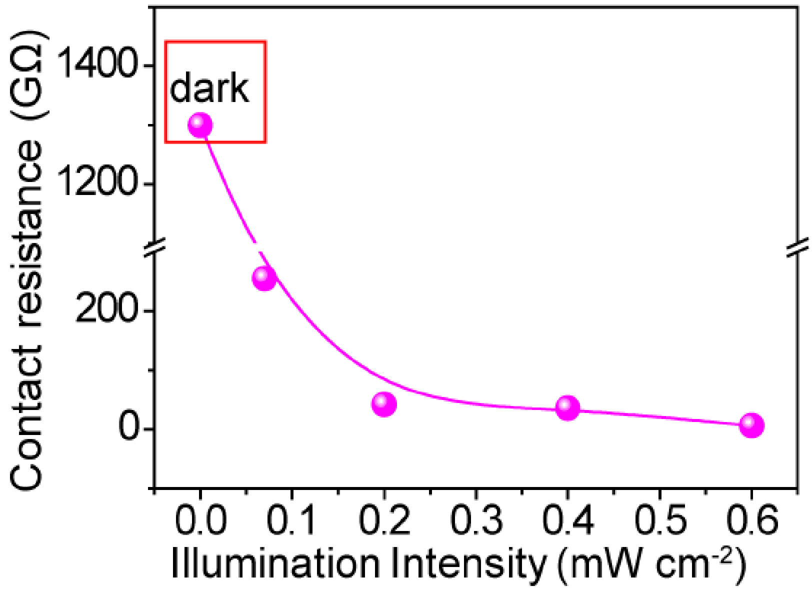

Supplementary Figure 17| Contact resistance of the device. The contact resistance in the dark and under different illumination densities (V_{GS} = 0 V).

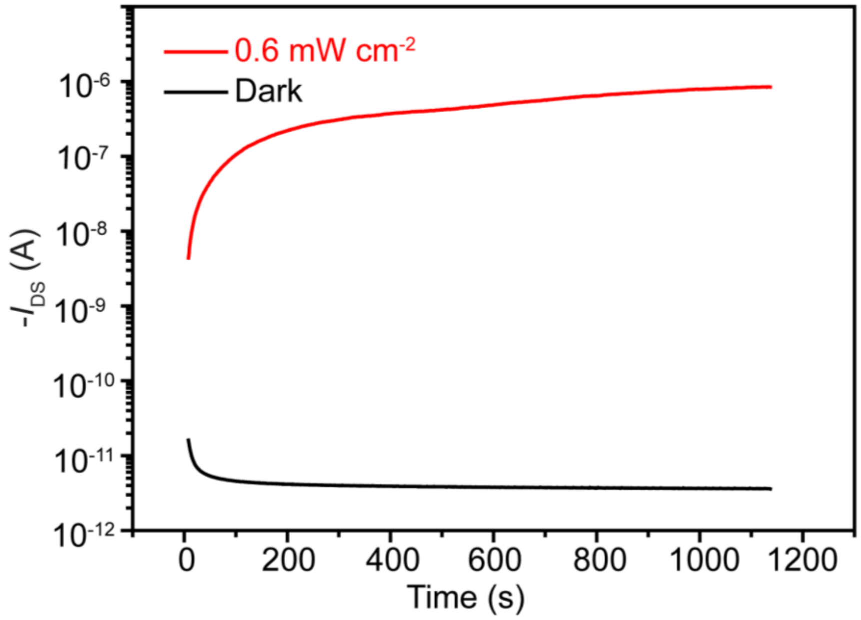

Supplementary Figure 18| The output current stability characterization. The output current in the dark and under illumination as a function of cycling time (V_{DS} = -100 V, V_{GS} = 0 V). The output current induced by illumination (0.6 mW cm^{-2}) was stable and the $I_{illumination}/I_{dark}$ ratio could be controlled around at least 10^4.

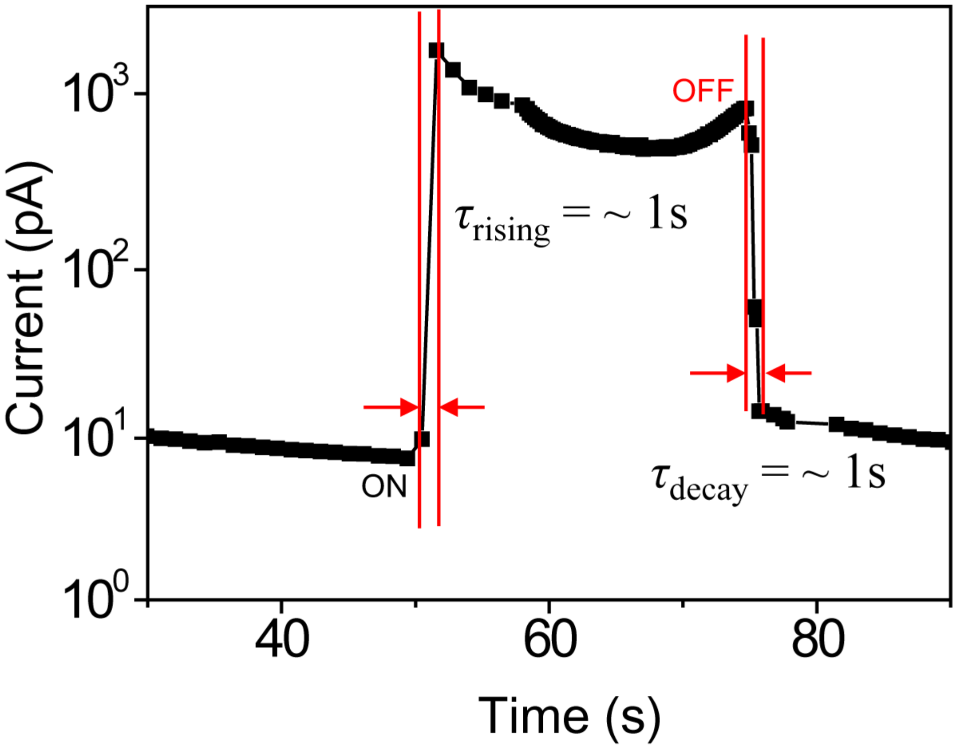

Supplementary Figure 19| The switching time of the three-terminal photodetector. The photoswitching time of τ_{rising} = ~ 1 s and τ_{decay} = ~ 1s.

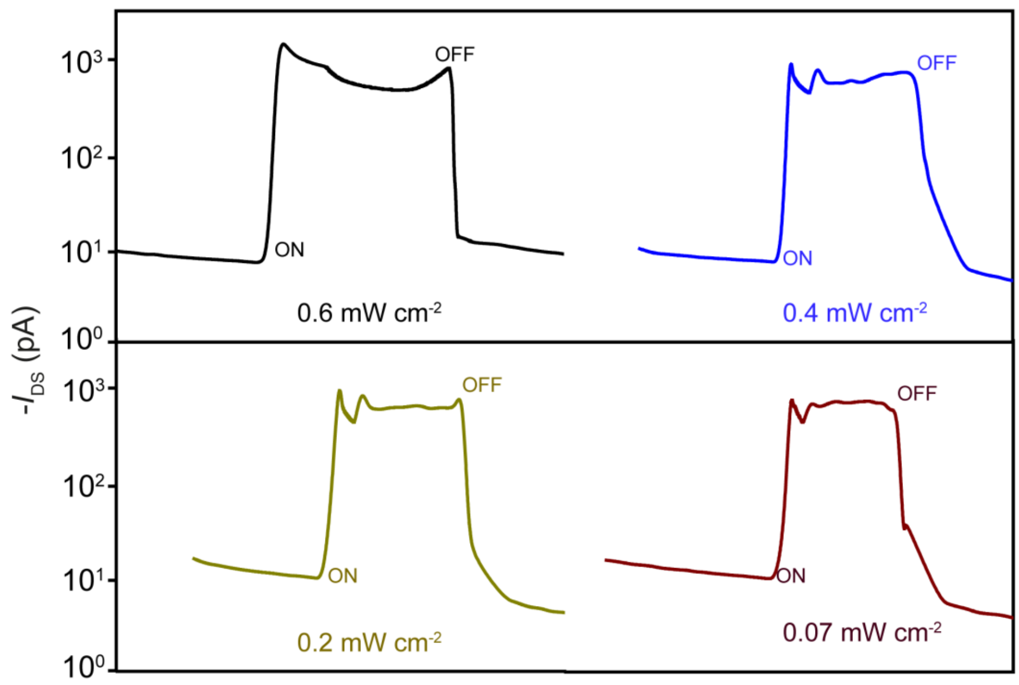

Supplementary Figure 20| The performance of the photodetector. The ratio of ON-OFF source-to-drain current ($-I_{DS}$) under different illumination intensity (V_{DS} = -100 V, V_{GS} = 0 V).

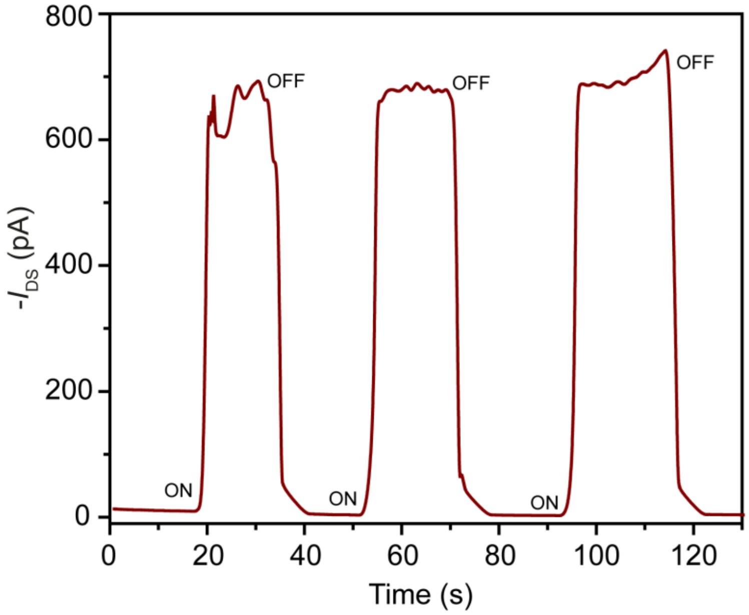

Supplementary Figure 21| The output performance of the photodetector. The source-to-drain current ($-I_{DS}$) recorded over time at a V_{DS} of -100 V and V_{GS} of 0 V, while turning the light on and off for three cycles.

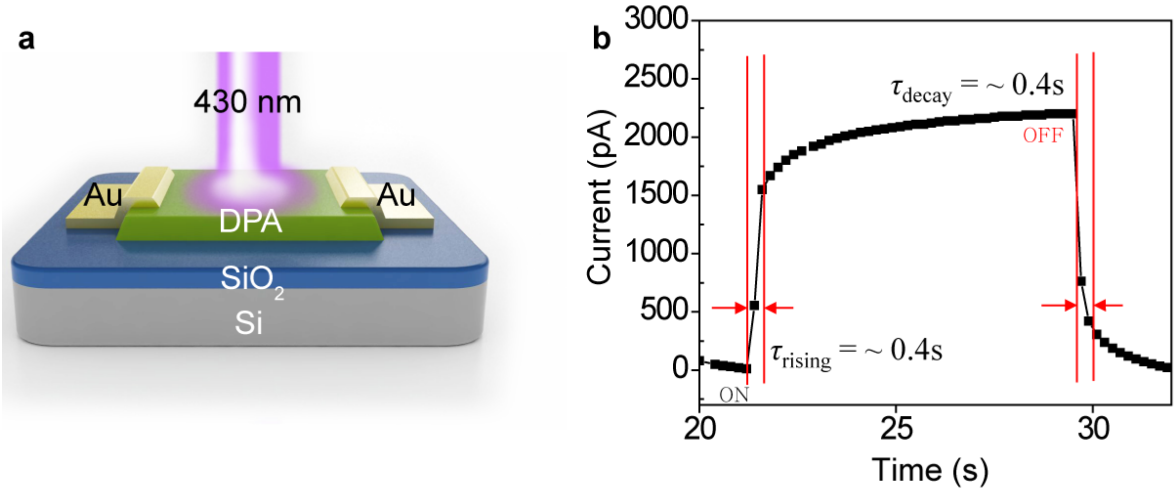

Supplementary Figure 22| The switching time of the two-terminal photodetector. (a) Schematic diagram of the two-terminal device. (b)The photoswitching time of τ_{rising} = ~ 0.4 s and τ_{decay} = ~ 0.4 s.
